# Cement-in-cement revision for Vancouver B2 periprosthetic femoral fractures: indications, surgical principles, and clinical outcomes — a narrative review

**DOI:** 10.1016/j.jor.2026.04.007

**Published:** 2026-04-20

**Authors:** Alessio Manco, Jacopo Vittori, Francesco Bosco, Norsaga Hoxha, Umberto Cottino, Federico Dettoni

**Affiliations:** a Department of Surgery, Università Degli Studi di Torino, Torino, Italy; b Department of Orthopaedics and Traumatology, Ospedale Cardinal Massaia Asti, Asti, Italy; c Department of Orthopaedics and Traumatology, G.F. Ingrassia Hospital Unit, ASP 6, Palermo, Italy; d Department of Precision Medicine in Medical, Surgical and Critical Care (Me.Pre.C.C.), University of Palermo, Palermo, Italy; e AO Ordine Mauriziano, Department of Orthopaedics and Traumatology, University of Torino, Largo Turati 62, 10128 Torino, Italy

**Keywords:** Cement-in-cement technique, Vancouver B2 fracture, Periprosthetic femur fracture

## Abstract

Vancouver B2 periprosthetic femoral fractures represent a major surgical challenge, particularly in elderly and frail patients. Revision with long uncemented stems remains the standard treatment, but it is technically demanding and associated with substantial surgical morbidity. The cement-in-cement technique has emerged as a less invasive alternative that may reduce operative burden in carefully selected cases. The aim of this narrative review was to critically evaluate the current evidence regarding the use of the cement-in-cement technique for the management of Vancouver B2 periprosthetic femoral fractures. A literature search was conducted using PubMed and Scopus to identify studies reporting indications, surgical principles, and clinical outcomes of this technique. Available evidence suggests that cement-in-cement revision is associated with complication and re-revision rates comparable to those of uncemented long-stem revision, while offering shorter operative times and similar implant survivorship. The cement-in-cement technique may be a viable option for selected patients undergoing revision hip arthroplasty, particularly when the distal cement mantle is well-fixed. However, current evidence remains limited, and further studies with larger cohorts and longer follow-up are required to better define indications and long-term outcomes.

**Level of evidence:**

Level IV

## 1 Introduction

The incidence of periprosthetic proximal femoral fractures (PPFFs) is increasing alongside the growing number of primary and revision hip arthroplasties.^1^^,^^2^ These fractures represent a severe complication associated with substantial morbidity, mortality, and risk of reoperation.^3^, ^4^, ^5^ Among them, Vancouver type B2 fractures—characterized by a loose femoral stem with preserved bone stock—pose a particular surgical challenge.^6^

Revision with long uncemented stems is widely considered the standard treatment, as it allows distal fixation and biological stability.^6^ However, this approach is technically demanding and may be associated with prolonged operative time, increased blood loss, and higher surgical morbidity, especially in elderly and frail patients.^7^ Furthermore, removal of a well-fixed cement mantle can be invasive and may further compromise femoral bone stock.^8^

In this context, preservation of the existing cement mantle through a cement-in-cement revision has emerged as a less invasive alternative for carefully selected Vancouver B2 fractures. By avoiding extensive cement removal, this technique may reduce operative burden while maintaining adequate mechanical stability.^8^

Despite growing interest in this approach, the available evidence remains limited and heterogeneous, and its role in the management of Vancouver B2 fractures has not yet been clearly defined. Clarifying the indications and outcomes of this technique is essential for optimizing surgical decision-making in this high-risk population.

The aim of this narrative review is to critically evaluate the current evidence on the cement-in-cement technique for the management of Vancouver B2 periprosthetic femoral fractures, with particular focus on indications, surgical principles, and clinical outcomes.

## 2 Methods

A narrative review of the current literature on the cement-in-cement technique for the management of Vancouver B2 periprosthetic femoral fractures was conducted. A structured search was performed using the PubMed and Scopus databases from database inception to January 2026. No restrictions were applied regarding study design or level of evidence. Articles were selected based on their relevance to the indications, surgical principles, clinical outcomes, and comparison with alternative revision strategies for the cement-in-cement technique. Studies were included if they reported clinical outcomes, surgical indications, or technical aspects of the cement-in-cement technique in the setting of Vancouver B2 periprosthetic femoral fractures. Exclusion criteria comprised studies not specifically addressing B2 fractures, reports lacking clinical or surgical detail, expert opinions without original data, and non-English language publications. This review was not conducted according to systematic review methodology, and no formal quality assessment of the included studies was performed.

## 3 Epidemiology and clinical impact

The incidence of revision hip arthroplasty is projected to increase substantially in the coming decades, with periprosthetic femoral fractures among the fastest-growing indications for revision surgery.^1^^,^^2^ These fractures impose a significant clinical burden, being associated with high mortality, complication rates, and functional decline. A multicenter study reported a one-month mortality of 9.3%, rising to 22.3% at one year, largely reflecting the advanced age and comorbidities of this population.^9^ Moreover, postoperative complications occur in nearly half of patients, and up to 60% fail to regain their pre-injury ambulatory status.

### 3.1 Anatomy and biomechanics relevant to B2 fractures

**Fig. 1:**
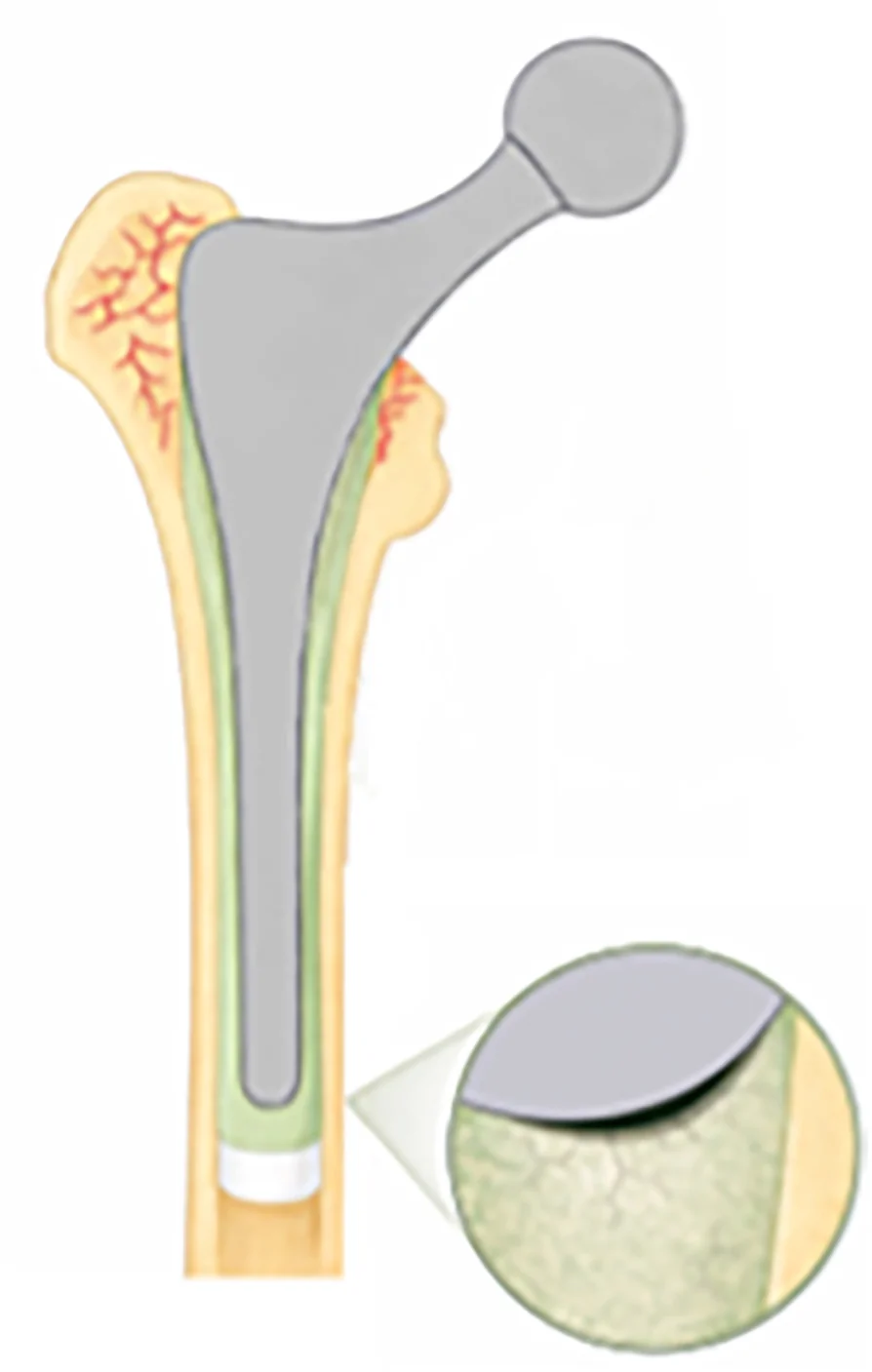
Schematic representation of femoral stem subsidence within the cement mantle, illustrating the generation of radial stresses at the bone–cement interface that may contribute to the development of Vancouver type B2 periprosthetic fractures.

**Fig. 2:**
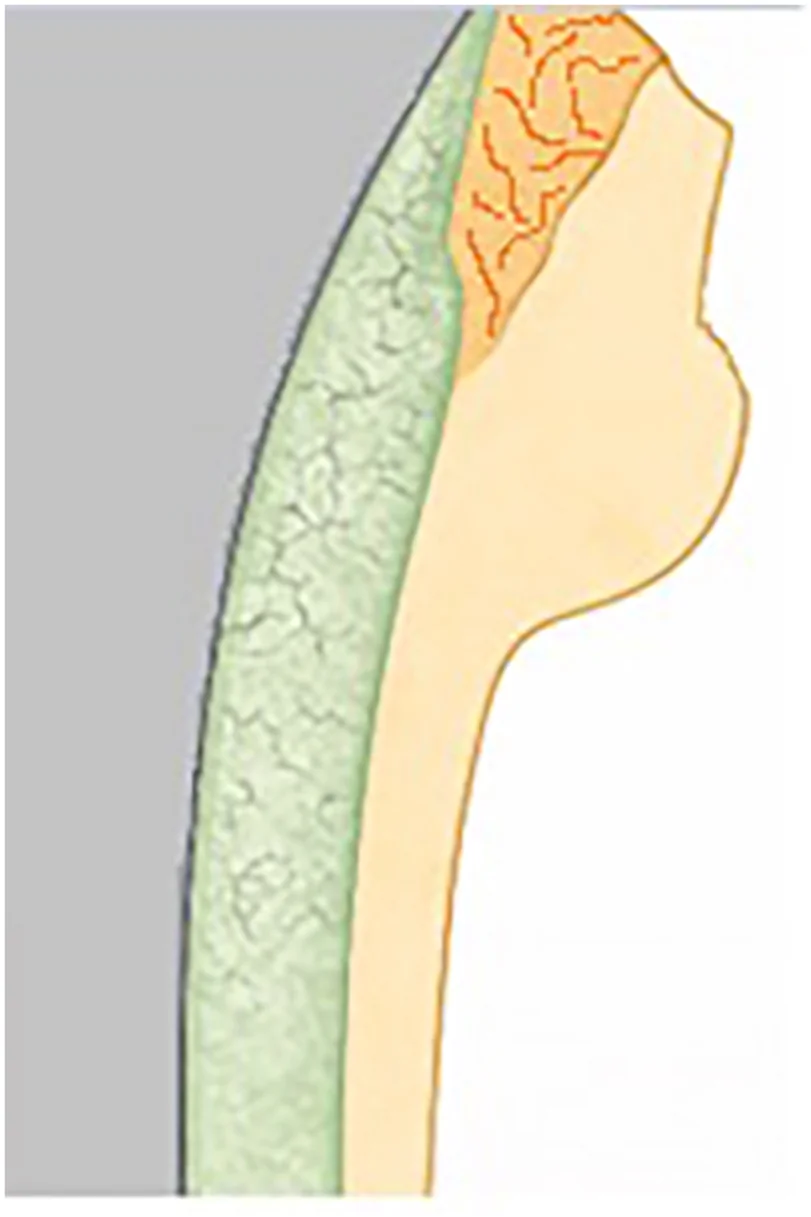
Microscopic view of fatigue microcracks within the cement mantle caused by cyclic loading, a process that progressively weakens the structural integrity of the cement and may lead to stem loosening.

Vancouver B2 fractures occur in the presence of a loose femoral stem with preserved bone stock and are strongly influenced by the mechanical behavior of the bone–cement–implant construct. In polished taper-slip stems, controlled subsidence within the cement mantle generates radial stresses that enhance fixation but may also concentrate forces on the femoral cortex, predisposing to fracture (Fig. 1).^10^ Progressive degeneration of the cement mantle plays a central role in stem loosening. Cyclic loading can induce fatigue microcracks, while debonding at the stem–cement interface promotes micromotion and further structural damage, ultimately resulting in axial and rotational instability (Fig. 2). 11, 12, 13 From a surgical perspective, preservation of a well-fixed distal cement mantle during revision may reduce operative time and limit femoral bone loss. When fracture reduction is achievable and the mantle remains stable, cement-in-cement revision represents a biomechanically sound option.^14^ Nevertheless, concerns persist regarding the biological impact of cement retention, particularly its potential effect on endosteal blood supply and fracture healing.^15^

## 4 Treatment options for Vancouver B2 fractures

Cementless long stems are commonly used for revision of Vancouver B2 periprosthetic fractures when reliable diaphyseal fixation is required. This approach enables distal mechanical stability and biological osseointegration but is technically demanding, particularly because cement removal may prolong operative time and increase surgical morbidity in frail patients.^16^ Early postoperative stem subsidence, especially with modular tapered designs, remains a recognized limitation of these implants.^17^ The cement-in-cement revision technique represents an established alternative when the distal cement mantle is well fixed and fracture reduction is achievable, thereby avoiding complete cement removal. The procedure involves bonding new cement to a properly prepared existing mantle, allowing implantation of a new stem while preserving femoral canal integrity.^18^ Its reduced surgical invasiveness may be particularly advantageous in elderly or medically complex patients, where minimizing operative time and perioperative complications is a priority.^14^ However, this technique is not universally applicable and requires careful patient selection, as inadequate cement fixation or poor fracture stability may compromise mechanical outcomes. Consequently, thorough preoperative assessment and intraoperative evaluation remain essential to ensure appropriate indication.

### 4.1 Combined cement-in-cement and adjunct fixation

**Fig. 3:**
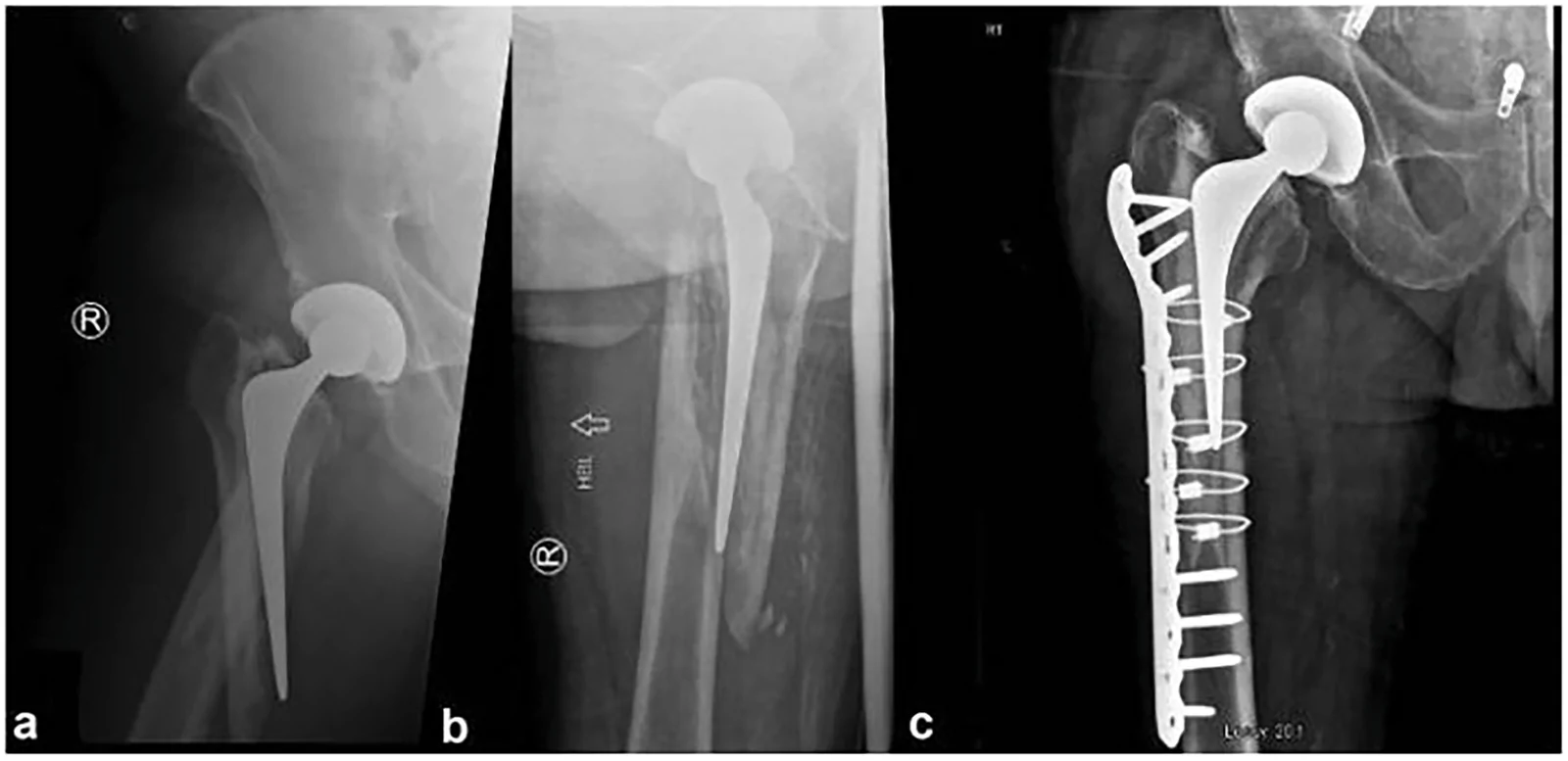
Anteroposterior radiograph of the periprosthetic fracture in a total hip arthroplasty (THA). (b) Vertical orientation of the cross table lateral radiograph. (c) The same patient after cement-in-cement with open reducution internal fixation (ORIF) revision to a short Exeter stem. Adapted from McCarthy et al..^22^

In more complex fracture patterns or when bone quality is poor, cement-in-cement revision can be combined with supplemental fixation to enhance construct stability. Cerclage wires, cables, and plates help control proximal fragments and improve load distribution around the retained cement mantle (Fig. 3).^14^^,^^19^ When substantial cortical defects are present, cortical onlay allograft struts may further increase structural support and reduce the risk of mechanical failure.^20^

### 4.2 Surgical technique

Patient selection for cement-in-cement revision relies on careful radiographic evaluation of the cement mantle to confirm its stability and exclude loosening at the bone–cement interface.^14^ Preoperative planning includes selecting a revision stem with a smaller diameter than the removed implant to allow adequate interposition of new cement within the existing mantle.^8^

The procedure is typically performed through an extended posterolateral approach. After removal of the loose femoral component, the fracture is exposed to assess distal and proximal cement fixation. When the mantle is confirmed to be well fixed, the fracture is anatomically reduced, and the canal is inspected intramedullary—optionally with a rigid arthroscope—to verify mantle integrity. The cement is then cleaned and thoroughly dried before introducing new cement.

The revision stem is inserted during the high-viscosity phase to optimize cement-to-cement bonding and minimize extrusion into the fracture site. A smaller stem is preferred to ensure an adequate cement mantle thickness, while partial cement removal with a high-speed burr may be performed when necessary. Supplemental fixation with cerclages, cables, or plate constructs is recommended to provide rigid fracture stability.^8^ In selected cases, a longer cement-in-cement stem may be used to bypass the fracture by at least 2.5 cortical diameters, with placement of a new cement restrictor.^21^

Immediate full weight-bearing is often permitted because of the stability provided by intramedullary cementation combined with adjunct fixation. Radiographic follow-up typically includes serial assessments, with consolidation frequently observed by six months.^8^ Nevertheless, limited follow-up and reports of late complications highlight the importance of continued surveillance.^8^^,^^22^ Failure should be suspected in the presence of persistent pain, loss of reduction, nonunion, refracture, or stem loosening.^8^^,^^22^

## 5 Results

Several studies have evaluated the cement-in-cement technique for the treatment of Vancouver B2 periprosthetic femoral fractures, consistently describing it as a viable option in carefully selected patients. The studies by Kennedy et al.^15^ and Klasan et al.^21^ provide the most relevant comparative data regarding clinical outcomes and survivorship.

Patient survivorship following Vancouver B2 fractures appears comparable between cement-in-cement and uncemented stem revision. Kennedy et al.^15^ reported no significant difference in survival in a cohort of 72 patients (29 cement-in-cement vs 43 uncemented; p = 0.572). Similarly, Klasan et al.,^21^ analyzing 101 patients (31 cement-in-cement, 70 uncemented), found comparable 5-year survivorship rates (62.5% vs 69.8%, p = 0.094).

In the study by Kennedy et al.,^15^ no postoperative dislocations were observed. Re-revision occurred in 5 of 29 patients (17.2%), including three subsequent periprosthetic fractures and two infections. No fracture non-unions were reported, and no perioperative deaths occurred within the cement-in-cement cohort, although deaths were recorded during follow-up for causes unrelated to surgery.

Klasan et al.^21^ reported one postoperative dislocation and a re-revision rate of 6.5% (2 of 31 patients), including one revision for dislocation and one DAIR procedure for deep infection. Two perioperative deaths (6.5%) were documented, while all fractures achieved radiographic union by six months.

Both cement-in-cement and uncemented modular stem revision demonstrate comparable implant survivorship. Kennedy et al.^15^ found no significant difference in revision-free survival at a mean follow-up of 4.1 years (p = 0.955). Likewise, Klasan et al.^21^ reported equivalent 5-year implant survival (93.5% vs 94.4%; p = 0.946).

Comparative studies report shorter operative times with the cement-in-cement technique. Kennedy et al.^15^ observed mean operative times of 140 min versus 160 min for cementless revision (p = 0.046), while Klasan et al.^21^ described an average reduction of approximately 45 min (p < 0.001).

Regarding blood loss, Kennedy et al.^15^ found no significant differences between groups in either transfusion requirements (p = 0.995) or perioperative hemoglobin variation (p = 0.402).

## 6 Discussion

The available evidence suggests that cement-in-cement revision is a reliable option for carefully selected Vancouver B2 periprosthetic femoral fractures, demonstrating implant survivorship and revision rates comparable to those of long cementless stems while offering the advantage of reduced surgical invasiveness and shorter operative times. These characteristics make the technique particularly attractive for elderly or medically frail patients.

Comparative studies support these findings. Kennedy et al.^15^ reported similar revision rates (18.6% vs 17.2%) and mean implant survival (93% vs 96.7%) between uncemented and cement-in-cement revision. Likewise, Da Assunção et al.^23^ observed a revision rate of 7.8% and a dislocation rate of 7.8% at a mean follow-up of three years, results consistent with those reported by Klasan et al.,^21^ who found a re-revision rate of 6.5% and a dislocation rate of 3.2%.

Biomechanically, the technique can provide effective load transfer when a well-fixed cement mantle is preserved, thereby limiting surgical trauma. However, long cementless stems remain preferable when the mantle is inadequate, fracture reduction is unstable, or diaphyseal fixation is required.^16^ Consequently, meticulous case selection is essential to minimize the risk of mechanical failure.

Potential limitations of cement-in-cement revision should also be considered. The technique is appropriate only when the existing mantle is continuous, stable, and free from loosening; otherwise, alternative revision strategies are recommended.^8^ Concerns regarding fracture healing persist, as residual cement near the fracture site may theoretically interfere with biological repair processes.^24^

The choice of revision strategy should therefore be individualized according to patient characteristics, fracture pattern, and cement mantle quality. In appropriately selected patients, cement-in-cement revision offers a less invasive alternative with outcomes comparable to other techniques, whereas cementless diaphyseal fixation remains preferable in younger or higher-demand individuals and in cases of compromised cement integrity.

Current evidence is limited by small, predominantly retrospective series and relatively short follow-up, while heterogeneity in indications and fixation methods restricts direct comparison with other revision strategies. Moreover, most available studies do not report confidence intervals or adjusted analyses, further limiting the strength and generalizability of the reported outcomes. Future prospective multicenter investigations are needed to better define indications, evaluate long-term implant survival, and establish standardized criteria for assessing cement mantle quality.

From a clinical perspective, cement-in-cement revision should be primarily considered in elderly or frail patients with a well-fixed mantle, where minimizing surgical burden is a priority.

## 7 Conclusions

Cement-in-cement revision may represent a reliable option for carefully selected Vancouver B2 periprosthetic femoral fractures, demonstrating complication and revision rates comparable to alternative revision strategies while offering the advantage of reduced surgical invasiveness. The technique appears particularly valuable in elderly or medically frail patients when a well-fixed cement mantle is preserved. Nevertheless, successful outcomes are highly dependent on appropriate patient selection and meticulous surgical execution. Further high-quality studies are required to better define indications and evaluate long-term outcomes.

## Guardian/patient's consent

Not Applicable.

## Ethics approval

Compliant with Helsinki.

## Consent

Not applicable.

## Code availability

Not applicable.

## Ethical statement

This study was conducted in accordance with the ethical standards of the institutional and/or national research committee and with the 1964 Helsinki Declaration and its later amendments or comparable ethical standards.

## Authors’ contributions

AM and JV contributed to the study conception and design. Material preparation and data collection were performed by AM, and NH. Data analyses were performed by NH and FB. The first draft of the manuscript was written by AM, NH, and JV, and all authors commented on previous versions of the manuscript. FB, UC, and FD assessed the scientific contents and the writing. All authors read and approved the final manuscript.

## Funding statement

No funding has been received for this study.

## Conflict of interest statement

On behalf of all authors, the corresponding author states that there is no conflict of interest.

## Data Availability

Available on request.
